# Prognostic Value of the Albumin-to-γ-glutamyltransferase Ratio for Gallbladder Cancer Patients and Establishing a Nomogram for Overall Survival

**DOI:** 10.7150/jca.49242

**Published:** 2021-05-13

**Authors:** Lejia Sun, Xindi Ke, Dongyue Wang, Huanhuan Yin, Bao Jin, Haifeng Xu, Shunda Du, Yiyao Xu, Haitao Zhao, Xin Lu, Xinting Sang, Shouxian Zhong, Huayu Yang, Yilei Mao

**Affiliations:** 1 Department of Liver Surgery, Peking Union Medical College (PUMC) Hospital, PUMC & Chinese Academy of Medical Sciences, Beijing, 100730, China.; 2 Peking Union Medical College (PUMC), PUMC & Chinese Academy of Medical Sciences, Beijing, 100730, China.

**Keywords:** albumin-to-γ-glutamyltransferase ratio, gallbladder cancer, prognostic value, nomogram.

## Abstract

**Purpose:** The albumin-to-γ-glutamyltransferase ratio (AGR), a novel inflammation-related index, has been reported to have prognostic importance in several malignancies but not yet in gallbladder cancer (GBC). This study intended to assess the prognostic value of AGR in GBC and to develop a nomogram based on AGR for predicting overall survival (OS) in GBC patients after surgery.

**Methods:** Medical records of 140 qualified GBC patients between July 2003 and June 2017 were retrospectively analyzed. The function “surv_cutpoint” in the R package “survminer” was implemented to discover the optimal cut-off value of AGR. A nomogram on the fundamental of Cox model was established in the training cohort and was internally validated using calibration curves, Harrell's concordance index, time-dependent AUC plots and decisive curve analyses.

**Results:** The optimal AGR cut-off value concerning overall survival was 2.050. Univariate and multivariate analyses demonstrated that AGR (HR=0.354, *P*=0.004), T stage (HR=3.114, *P*=0.004), R0 resection (HR=0.448, *P*=0.003), BMI (HR=0.470, *P*=0.002) and CA19-9 (HR=1.704, *P*=0.048) were independent predictors for OS. The nomogram combining these prognostic factors showed considerable prognostic performance in term of consistency, discrimination and net benefit.

**Conclusion:** AGR has independent prognostic value for OS in GBC patients receiving surgery. A nomogram incorporating AGR, T stage, R0 resection, CA19-9 and BMI achieved enhanced prognostic ability.

## Introduction

As the fifth most common malignancy of the gastrointestinal tract, gallbladder cancer (GBC) constitutes the majority of carcinoma that originates from the extrahepatic biliary tract 1. Due to its aggressive features, pronounced tendency for early lymph node metastases and difficulties in early diagnosis, GBC patients usually display poor prognosis 2, 3. Currently, cholecystectomy remains the only curative intended treatment option for GBC 4. As a result of the widespread application of laparoscopic cholecystectomy, prognosis has improved in recent years 5. Unfortunately, most of GBC patients are diagnosed at a late stage and thus not eligible for radical surgery 1, 2. Although the prevalence of GBC is low, GBC is attracting increasing attention because the 5-year OS rate has decreased in recent years according to the latest data in China 6.

Tumor-Node-Metastasis (TNM) staging system is widely applied to evaluate the clinical outcome in GBC patients, but it has been recognized that the prognosis is varied in patients with the same TNM stage. Other pathological characteristics such as tumor differentiation and tumor size are also applied in the estimation of survival of GBC patients. However, none of them have taken patient-related factors, such as nutritional status and inflammation response, into consideration. In recent years, there has been accumulating evidence that inflammation facilitates tumor progression and correlates with the prognosis of cancer patients 7, 8. Inflammation markers and inflammation-related ratio indices, such as platelet-to-lymphocyte ratio (PLR) 9-11, neutrophil-to-lymphocyte ratio (NLR) 9, 10, 12-14, monocyte-to-lymphocyte ratio (MLR) 10, 15 and fibrinogen-to-albumin ratio (FAR) 16-18, have been increasingly investigated for their prognostic value in GBC as well as other types of cancer. However, these parameters have some limitations, and their benefit in clinical application remains to be verified. Therefore, there is still an urgent need to seek novel prognostic factors for GBC as well as other malignancies.

Albumin (ALB) and γ-glutamyltransferase (GGT) are factors for evaluating liver function and inflammation status. ALB, a protein with multiple functions, is synthesized by the liver. Hypoalbuminemia is an indicator of liver dysfunction, malnutrition, systemic inflammation and some other diseases 19, 20. Emerging evidence has shown that serum ALB and ALB-based ratio indices are independent prognostic factors for GBC and several other malignancies 18, 21, 22. GGT is an essential enzyme that plays a role in glutathione metabolism 23. Quite a few researches have revealed that an increased level of GGT is related to high cancer risk and poor prognosis 24. Based on previous researches, it is reasonable to hypothesize that the ALB-to-GGT ratio (AGR), a combination of the two parameters, can be a potent prognostic factor for GBC patients. In fact, Jing et al proposed AGR for the first time in 2017 and showed that AGR was a predictor for the prognosis of intrahepatic cholangiocarcinoma patients 25. Later, AGR and the GGT-to-ALB ratio were demonstrated as independent prognostic factors for both recurrence-free survival and OS in hepatocellular carcinoma and pancreatic ductal adenocarcinoma patients, respectively, after radical surgery 26, 27.

Currently, no study has focused on the prognostic value of AGR in GBC patients. We aimed to evaluate the prognostic significance of AGR for GBC and sought to construct a new nomogram on the basis of AGR for predicting OS of GBC patients after surgery.

## Methods

### Patients

All GBC patients at Peking Union Medical College Hospital (PUMCH) between July 2003 and June 2017 were retrospectively reviewed. The inclusion criteria were listed as follows: 1) pathologically proven gallbladder cancer; 2) no other malignant tumors except GBC; 3) patients receiving surgical resection; and 4) patients without antitumor treatment before or during the surgery. The exclusion criteria included: 1) patients with incomplete follow-up data; 2) patients without complete measurement of ALB or GGT; 3) patients who underwent preoperative albumin transfusion; 4) patients with active inflammation diseases; and 5) patients with perioperative mortality. A total of 140 eligible gallbladder cancer patients were enrolled and their data were analyzed in this study.

### Ethics statement

This study was supported by the Medical Ethics Committee of PUMCH. Signed informed approval was obtained from all patients. Our study was accompanied with the ethical criteria of the Declaration of Helsinki.

### Data collection and definition

All medical documents of GBC patients at our hospital were collected for demographic and clinicopathologic data, including age, sex, body mass index (BMI), tumor number, maximal tumor diameter, tumor differentiation, TNM stage, R0 resection, comorbidities, CA19-9, ALB and GGT, hospital stay, bleeding volume during surgery and postsurgical complications. The clinical stage was classified according to the 8th edition of the American Joint Committee on Cancer (AJCC) TNM staging system for GBC. In order to collect preoperative hematological parameters, such as CA19-9, ALB and GGT, blood samples before breakfast were obtained within 5 days before the operation. The index AGR was defined as the level of serum ALB level (g/L) divided by the level of serum GGT level (U/L). Multiple imputation was utilized for handling missing values of several clinicopathological characteristic, including BMI (19 missing data), tumor size (6 missing data), and CA19-9 (9 missing data).

### Follow-up

Patients are required to return to the hospital every 3 months in the first 2 years after the surgery and every 6 months in the third year, and thereafter once a year for patients who have no signs of recurrence. Telephone calls were conducted for follow-ups to obtain the treatment information and living status if patients did not visit our hospital on schedule. The primary endpoint was OS, which was determined as the duration from the operation to death or the last follow-up.

### Statistical analysis

The function “surv_cutpoint” in the R package “survminer” was applied to determine the optimal cut-off value of AGR, NLR, PLR, MLR and FAR. Quantitative variables are expressed as median and range, while number and percentage were reported for categorical variables. Comparisons of clinicopathological characteristics were performed via the Mann-Whitney U test for quantitative variables, and the Pearson χ*^2^* test or Fisher's exact test for categorical variables, as appropriate. The Kaplan-Meier method and the log-rank test were performed for survival analyses. Cox univariate and multivariate analyses were performed without violating the Proportional Hazards Assumption to analyze independent risk factors. The predictive accuracy of AGR was also compared with other inflammation-related indices, including MLR, PLR, NLR and FAR, by Harrell's concordance index (C index) and the time-dependent area under ROC curve (AUC) plot. The entire cohort was randomly divided into a training cohort (n=80) and a validation cohort (n=60). A nomogram integrating independent prognostic factors associated with OS was established in the training cohort. The performance of the nomogram was assessed in both the training and validation cohorts by calibration curves, C index, time-dependent AUC plots and decisive curve analyses (DCA). Statistical analyses were performed with Statistical Product and Service Solutions Software 26.0 (IBM Corporation, Armonk, NY, USA) and R software version 3.6.2 (http://www.r-project.org/). For all tests and analyses, a *P* value less than 0.05 was considered to be statistically significant.

## Results

### Baseline characteristics of the patients

In total, 140 qualified GBC patients were enrolled in the study. The last follow-up was conducted in February 2020. The median follow-up period was 21.5 months (range 1-141). 96 (68.57%) patients were confirmed dead at the time of the last follow-up. The median OS was 21 months, and the 1-, 3- and 5-year OS rates were 64.0%, 36.5% and 28.6%, respectively.

Of the entire cohort, 82 (58.6%) were females and 58 (41.4%) were males. The patients were 29-85 years of age with the median age as 64 years old. The median tumor size was 2.75cm (range 0.2-13.0) and there were 57 (42.1%) patients whose tumors were large than 3 cm. According to the 8th edition of AJCC TNM staging system, 4 (2.9%), 13 (9.3%), 12 (8.6%), 45 (32.1%), 45 (32.1%) and 21 (15.0%) patients were classified as stage 0, I, II, IIIA, IIIB and IV, respectively. The median levels of ALB and GGT were 41 g/L (range 27-50) and 40.5 U/L (range 12.0-1807.0), respectively. Detailed baseline characteristics of all participants were summarized in Table S1, and clinicopathological features of the training and validation cohorts were also presented.

### Relationship between AGR and clinicopathological characteristics

The cut-off value of AGR was determined as 2.050. Patients were stratified by the value of AGR into two groups: the high-risk group (AGR≤2.05, n=108) and the low-risk group (AGR>2.05, n=32). The relationship between AGR and other clinicopathological characteristics is presented in Table 1. The low-risk group was significantly associated with less advanced T stage (*P*<0.001), N stage (*P*=0.020), TNM stage (*P*=0.001), R0 resection (*P*=0.014), absence of jaundice (*P*=0.005), normal levels of CA19-9 (*P*<0.001), ALB (*P*=0.009) and GGT (*P*<0.001). It was also indicated that a high-risk level of AGR was correlated with high levels of NLR (*P*=0.022), MLR (*P*=0.001), PLR (*P*=0.001) and FAR (*P*<0.001). It seemed that AGR was not related to histopathological features such as tumor size (*P*=0.155) or tumor differentiation (*P*=0.141).

### Comparison of AGR with NLR, PLR, MLR and FAR

Inflammation-related indices have been increasingly investigated in cancer, and there are a few studies concerning the prognostic value of inflammation-related parameters in GBC. Among them, ratio indices, such as PLR, NLR, MLR and FAR, have been reported to be adverse prognostic factors for GBC 9, 10, 12, 15, 16. Therefore, we were interested in the predictive accuracy of AGR compared with these inflammation-related indicators.

The cut-off values of NLR, MLR, PLR and FAR were 1.734, 0.211, 159.0, and 0.084, respectively. More dismal prognosis was observed in patients with high levels of NLR (13 months vs. 65 months, *P*<0.001, Figure S1A), MLR (10 months vs. 40 months, *P* <0.001, Figure S1B), PLR (8 months vs. 34 months, *P* <0.001, Figure S1C) and FAR (13 months vs. 49 months, *P*<0.001, Figure S1D), respectively. The time-dependent AUC plots showed that the predictive accuracy of AGR was no less than that of NLR, MLR, PLR and FAR, especially during 24-36 months after surgery (Figure S2). The C index of AGR for OS prediction (0.618, 95% CI: 0.573-0.663) was also comparable to that of NLR (0.626, 95% CI: 0.583-0.669), MLR (0.630, 95% CI: 0.581-0.679), PLR (0.623, 95% CI: 0.572-0.674,) and FAR (0.627, 95% CI: 0.574-0.680).

### Prognostic significance of AGR for short-term and long-term outcomes

A low level of AGR was correlated with poor short-term outcomes: patients with a low level of AGR tended to spend more days in hospital (16 days vs. 11 days, *P*<0.001, Table 1) and suffered from more bleeding during surgery (200 mL vs. 80 mL, *P*=0.008, Table 1), and postsurgical complications were also more frequently present in the high-risk group (26.9% vs 6.3%, *P*=0.014).

**Figure 1:**
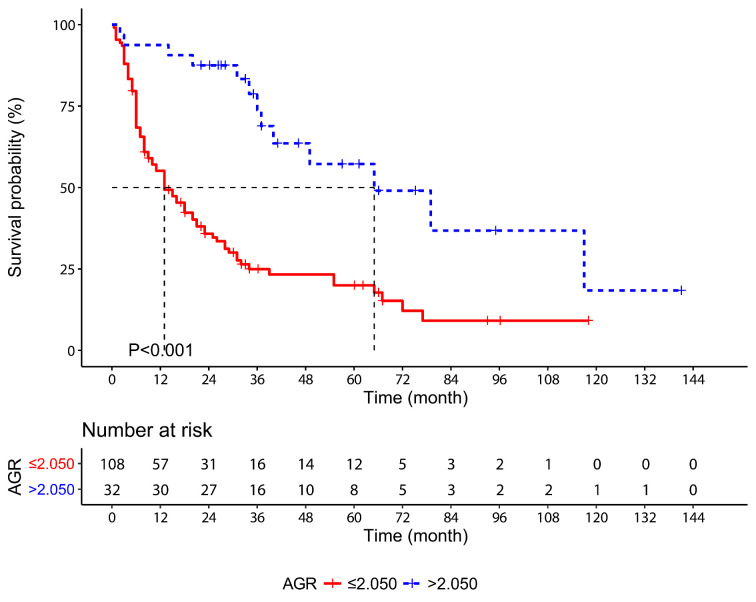
Kaplan-Meier survival curves for overall survival in gallbladder cancer patients stratified according to AGR. Abbreviations: AGR, albumin-to-γ-glutamyltransferase ratio.

According to the Kaplan-Meier survival curves, the median OS of the AGR high-level patients was 52 months longer than the AGR low-level patients (65 months vs. 13 months, *P*<0.001, Figure 1). The 1-, 3- and 5-year OS rates were significantly higher in the low-risk group than the high-risk group (93.8%, 73.8% and 57.2% vs. 55.1%, 25.0% and 20.0%, respectively, P<0.001).

Univariate analysis revealed that a high level of AGR was a significantly favorable factor for OS (HR=0.286, *P*<0.001, Table 2). In addition, high BMI (HR=0.548, *P*=0.005), poor tumor differentiation (HR=1.663, *P*=0.015), advanced T stage (T3-T4, HR=5.798, *P*<0.001), N stage (N1-N2, HR=2.643, *P*<0.001), R0 resection (HR=0.276, *P*<0.001), presence of jaundice (HR=2.074, *P*=0.004), high levels of CA19-9 (HR=3.421, *P*<0.001), NLR (HR=2.988, *P*<0.001), MLR (HR=2.387, *P*<0.001), PLR (HR=2.324, *P*<0.001) and FAR (HR=2.720, *P*<0.001) were also identified as predictors for OS.

Multivariate analysis revealed that a high level of AGR (HR=0.354, *P*=0.004, Table 2) was an independent favorable factor for OS. High BMI (HR=0.470, *P*=0.002), advanced T stage (HR=3.114, *P*=0.004), R0 resection (HR=0.448, *P*=0.003) and an elevated level of CA19-9 (HR=1.704, *P*=0.048) were also independent prognostic factors. Notably, NLR (HR=1.261, *P*=0.502), MLR (HR=0.800, *P*=0.457), PLR (HR=1.146, *P*=0.618), and FAR (HR=1.153, *P*=0.561) failed to be independent predictors in multivariate analysis, suggesting that AGR could be a better prognostic factor than these inflammation-related indices.

### Prognostic nomogram integrating AGR and other prognostic factors

**Figure 2:**
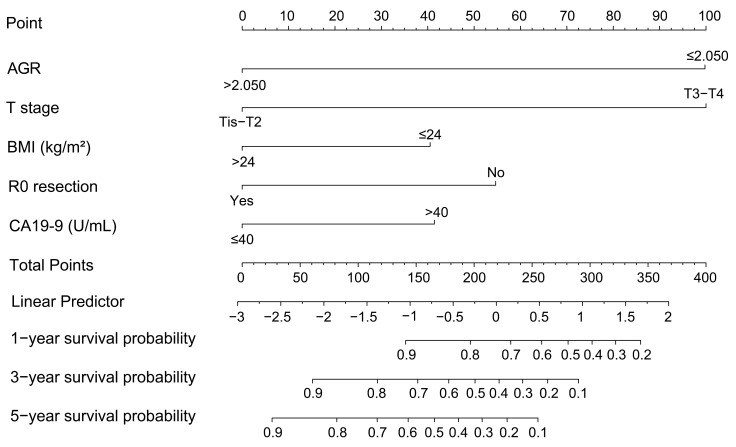
Nomogram based on AGR, T stage, R0 resection, BMI, and CA19-9 for predicting overall survival. Abbreviations: AGR, albumin-to-γ-glutamyltransferase ratio; BMI, body mass index; CA19-9, carbohydrate antigen 19-9.

**Figure 3:**
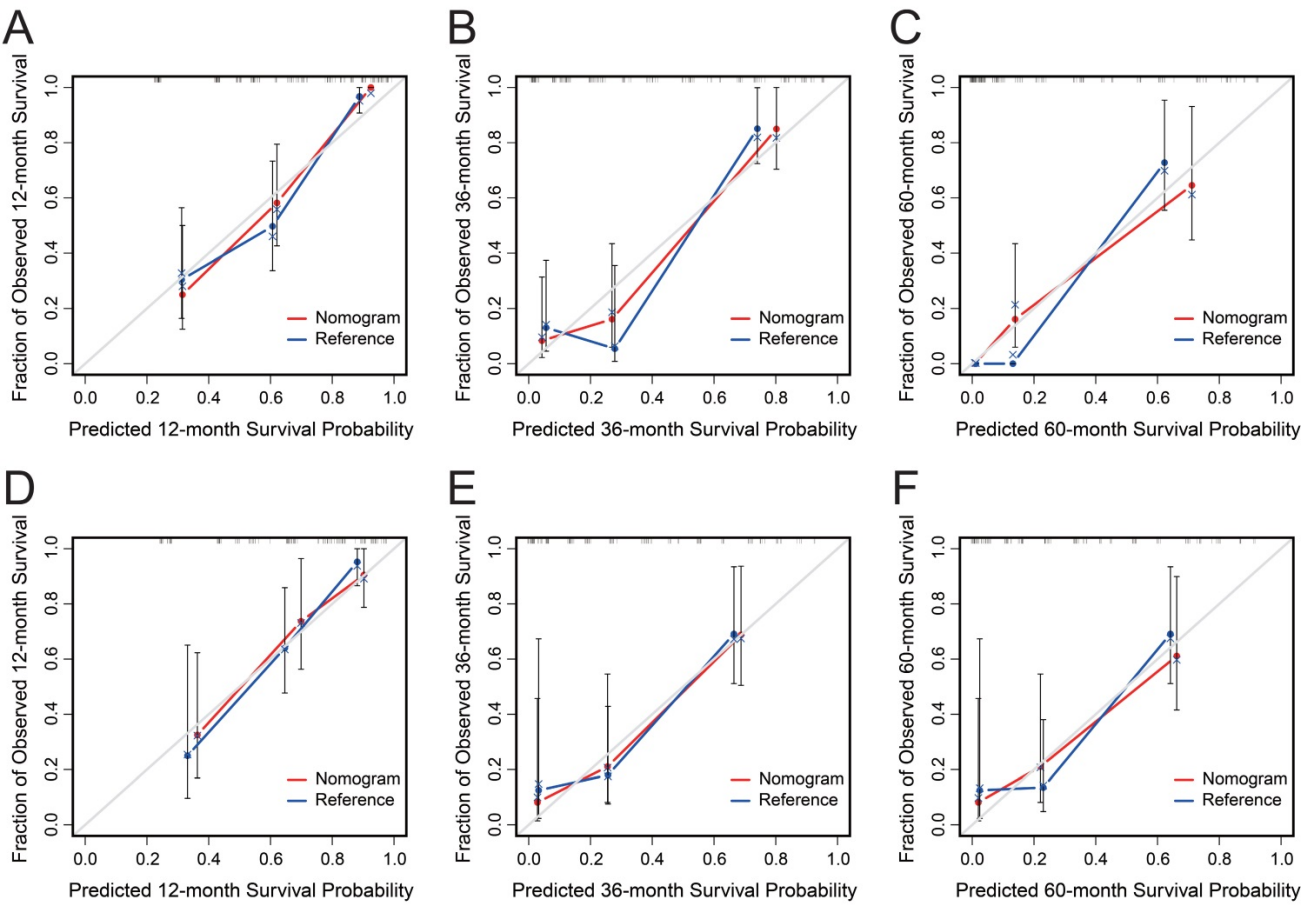
Calibration curves of the nomogram (red) and the reference model (blue) for 1-, 3- and 5-year overall survival of the training cohort (A-C) and the validation cohort (D-F). The x-axis represents nomogram predicted probability of survival, and the y-axis is the actually observed survival probability.

**Figure 4:**
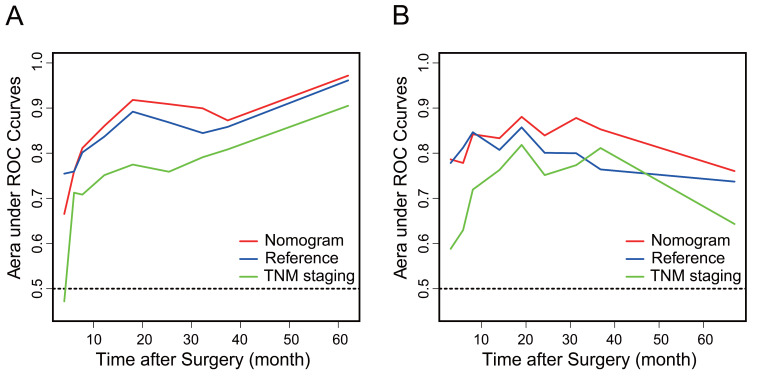
Time-dependent area under ROC curves of the nomogram (red), the reference model (blue) and the TNM staging system (green) in the training cohort (A) and the validation cohort (B).

AGR, T stage, R0 resection, BMI and CA19-9, which were independent prognostic factors for OS revealed by Cox regression analysis, were combined to develop a predictive nomogram for OS (Figure 2). To classify the contribution of AGR to the predictive nomogram, a similar model comprised of T stage, R0 resection, BMI and CA19-9 was set as the reference. The consistency of the nomogram was illustrated by calibration curves. The predicted lines overlapped well with the diagonal lines, suggesting that the nomogram-predicted OS was in good agreement with actually observed OS of GBC patients (Figure 3). Compared with the reference model, the nomogram showed better consistency, although such superiority was subtle in 1- and 3-year calibration curves of the validation cohort. The C index of the nomogram in the validation cohort was 0.762 (95% CI: 0.684-0.840), higher than that of the reference model (0.744, 95% CI: 0.662-0.826) and the TNM staging system (0.689, 95% CI: 0.603-0.775). In the training cohort, the C index of the nomogram (0.787, 95% CI: 0.721-0.853) was also higher than that of the reference model (0.771, 95% CI: 0.705-0.837) and the TNM staging system (0.694, 95% CI: 0.619-0.769). Time-dependent AUC plots of both the training and validation cohorts were also plotted, revealing that the nomogram had better discriminative ability compared with the reference model and the TNM staging system (Figure 4). To assess the clinical application value of the nomogram, DCA was conducted to compare the net benefit of the nomogram with the reference model and the TNM staging system (Figure S3). The DCA plots revealed that the nomogram yielded more net benefit 1 and 3 years after surgery across a wide range of threshold probability, suggesting that the nomogram could be more efficacious in clinical practice.

### Comparison with previous nomograms

The current nomogram was compared with two of the previous nomograms: Bai et al constructed a nomogram integrating the presence of jaundice, CA19-9, R0 resection and TNM stage 28; Deng et al developed a nomogram based on the lymphocyte/monocyte ratio, tumor differentiation, TNM stage and radical surgery 29. We conducted the comparison in the entire cohort via calibration curves, C index, time-dependent AUC plots and DCA. The 1-, 3- and 5-year calibration curves indicated that our nomogram had more superior consistency than the other two (Figure S4). The C index of our nomogram was 0.780 (95%CI: 0.731-0.829), higher than that of Bai's (0.751, 95%CI: 0.700-0.802) and Deng's models (0.752, 95%CI: 0.703-0.801). The time-dependent AUC plot also revealed that our nomogram achieved better predictive accuracy 10 months after the surgery than the other two nomograms (Figure S5). Although no obvious superiority was suggested by the 1-year DCA plot, the 3- and 5-year DCA plots indicated that our nomogram generated more net benefit across a wide range of threshold probability (Figure S6).

### Risk stratification model and subgroup analysis

**Figure 5:**
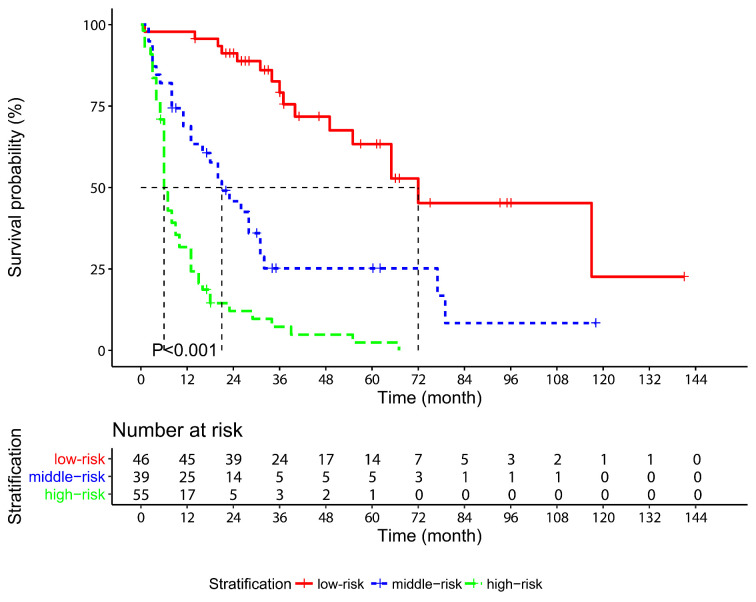
Kaplan-Meier survival curves for overall survival in gallbladder cancer patients stratified according to the risk stratification model based on the nomogram.

A novel stratification model was developed on the fundamental of the nomogram: each patient was classified into the low-risk (total points: 0-195.9, n=46), middle-risk (total points: 196.0-289.9, n=39) or high-risk (total point: 290.0-336.4, n=55) group depending on the total points. The median OS of the low-risk, middle-risk and high-risk groups was 72, 21 and 6 months, respectively (*P*<0.001, Figure 5). According to the year in which the operation was conducted, patients were divided into two subgroups: 2003-2012 (n=61) and 2013-2017 (n=79). Kaplan-Meier survival curves displayed that the OS of the patients was well distinguished according to the risk stratification model in both the 2003-2012 subgroup (*P*<0.001, Figure S7A) and the 2013-2017 subgroup (*P*<0.001, Figure S7B). Similar results were also observed in the survival curves of AGR in both the subgroups (Figure S7C-D).

### Nomogram-predicted survival probability of patients at the same TNM stage

The AJCC TNM staging system is a useful method for clinical practitioners, but it fails to provide precise prognostic information, especially for patients at an advanced TNM stage. To investigate whether our nomogram could distinguish the different outcomes of GBC patients classified as the same TNM stage, histograms of nomogram-predicted 1-, 3- and 5-year survival probability of patients at stage IIIA and IIIB were plotted. The predicted survival probabilities of patients in stage IIIA were quite different (Figure S8A); patients at stage IIIB generally had more dismal long-term outcomes, yet the nomogram-predicted survival still varied (Figure S8B). As for TNM stage I, II and IV, considerable differences in the nomogram-predicted survival probabilities were also discovered.

## Discussions

ALB, synthesized by liver, has multiple intracorporal functions, such as maintaining intravascular colloid pressure and facilitating the transportation of multiple substances 30. The level of serum ALB is a common indicator of nutritional status and liver function. ALB also serves as an antioxidant that contributes to the elimination of reactive oxygen and nitrogen species in systemic inflammation 31, 32. There is also evidence that ALB is involved in cellular signaling pathways, such as suppressing carcinogenesis by reducing the phosphorylation of Rb protein 33. Therefore, a low level of ALB represents impaired protection against tumors. GGT is a key enzyme essential for the metabolism of glutathione, which is an important protective substance for reducing oxidative stress 23. It is widely expressed in various organs and tissues especially hepatocytes and cholangiocytes 23. An elevated level of serum GGT activity is usually a reflection of hepatic and biliary diseases. More importantly, a high level of GGT also represents intense oxidative stress and a high risk of cancer 24, 34, and it is significantly associated with the prognosis of various malignancies 35-38. Combined with ALB and GGT, AGR is not merely an indicator of liver function and nutritional status, but also an inflammation-related index that represents the host inflammation response to tumors and has potential prognostic value.

In this study, we identified AGR, a novel inflammation-related ratio index defined by the preoperative levels of ALB and GGT, as an independent prognostic factor for GBC. As far as we know, this is the first study concerning the prognostic value of AGR for GBC. AGR was associated with a series of clinicopathological characteristics, such as T stage, N stage, TNM stage, R0 resection, presence of jaundice, and CA19-9. A low level of AGR was also found to be correlated with high levels of NLR, MLR, PLR and FAR. A high level of AGR indicated a better short-term outcome and longer OS. AGR high-level patients had evidently higher 1-, 3-, and 5-year survival rates than AGR low-level patients. AGR was combined with the other four independent prognostic factors, including T stage, R0 resection, CA19-9 and BMI, to establish and validate a predictive nomogram for 1-, 3, and 5-year survival probability. The nomogram achieved considerable prognostic performance in term of consistency, discrimination as well as net benefit, and AGR was proved to make real contributions to the predictive ability of the nomogram. The current nomogram also showed better consistency, discrimination and more net benefit compared with two of the previously established prognostic nomograms for GBC 28, 29. The stratification model based on the nomogram distinguished clearly the prognoses of patients in different risk groups. Subgroup analysis indicated that the long span did not compromise the predictive performance of the nomogram. The nomogram-predicted survival probability showed conspicuous heterogeneity even within the same TNM stage, suggesting that our nomogram stratified the prognosis of GBC patients receiving surgery better than the AJCC TNM staging system.

Although widely applied, the AJCC TNM staging system has intrinsic shortcomings because it only takes the conditions of primary tumor, lymph nodes and metastasis into consideration. Histopathological characteristics, such as tumor type, tumor number and tumor differentiation, also serve as indicators for the prognosis of cancer patients. So far, some prognostic nomograms that predict the survival of patients with GBC have been constructed. Although individualized prognostic information could be provided, many of them were mainly dependent on tumor-related characteristics and the therapy received 28, 39-44. Detailed computed tomography findings were also included in the development of prognostic nomograms, but still focused on tumor-related factors 45, 46. However, there is growing consensus that not only tumor intrinsic properties but also patient-related factors are closely relevant to survival prognosis of cancer patients 47. Therefore, currently available prognostic methods merely dependent on pathological features of tumor are far from comprehensive. Recently, Yadav et al proposed a novel staging system for gallbladder cancer integrating Eastern Cooperative Oncology Group (ECOG) score and the level of serum alkaline phosphatase 47; some of systemic inflammatory biomarkers were taken into consideration when establishing prognostic nomograms 10, 29. Our study, which investigated the prognostic value of the novel inflammation-related marker AGR and combined it with other independent prognostic factors to construct a nomogram for the prognosis of patients with resected GBC, was another beneficial attempt. With new prognostic models for GBC emerging, further studied are needed to evaluate and compare the efficacy of such models.

It is now clear that chronic inflammation, which leads to repeated tissue damage and restoration, is closely relevant to the progression and prognosis of various kinds of cancer 7, 8. Inflammation markers are drawing increasing attention for their prognostic value in recent years. Our study also showed that AGR had comparable prognostic accuracy with previously investigated inflammation-related markers including NLR, PLR, MLR and FAR. The integrated index AGR reflects the host inflammation and immunity status, and thus provides more valuable prognostic information from the patients' perspective. Therefore, AGR can serve as a potent prognostic factor alone or combined with other factors, such as pathological characteristics and tumor biomarkers, which was demonstrated in our study. Because AGR can be easily obtained from a routine liver function test, it can be conveniently applied in clinical setting and may be more beneficial for predicting the prognosis of patients without complete pathological features for lack of eligibility to surgery.

However, there are several shortcomings that should be addressed in our study. First of all, this is a retrospective study conducted in a single center in China and thus selection bias may be inevitable. Whether the cut-off value of AGR is optimal for other areas of the world and ethnicities remains to be confirmed. Second, only 140 GBC patients after surgery were enrolled in this study. Thus, the sample size is relatively small and the study lacks an external validation. Last but not least, the majority of GBC patients are not eligible for surgical resection, but these patients were not included in our study. Taking these limitations into consideration, large-scale, multi-center and prospective studies are required to verify our conclusions in GBC patients of different ethnicities and receiving other modalities of treatment.

In conclusion, our study demonstrated the prognostic value of AGR, a novel inflammation-related index, in GBC patients after surgery. AGR was demonstrated to be an independent prognostic factor for gallbladder cancer. The nomogram integrating AGR, T stage, R0 resection, BMI and CA19-9 was established as a prediction model, and was shown to have considerable predictive ability. The integration of AGR into the nomogram improved the predictive performance of the nomogram, suggesting that current prognostic methods would be promoted if combined with AGR.

## Supplementary Material

Supplementary figures and table.

## Figures and Tables

**Table 1:** Correlation between AGR and clinicopathological characteristics.

Variable	Classification	AGR ≤ 2.05 (n=108)	AGR > 2.05 (n=32)	*P*
Sex	Female	60 (55.6%)	22 (68.7%)	0.183
	Male	48 (44.4%)	10 (31.3%)	
Age	≤65	60 (55.6%)	20 (62.5%)	0.486
	>65	48 (44.4%)	12 (37.5%)	
BMI (kg/m^2^)	≤24	60 (55.6%)	16 (50.0%)	0.580
	>24	48 (44.4%)	16 (50.0%)	
Tumor size (cm)	≤3	59 (54.6%)	22 (6878%)	0.155
	>3	49 (45.4%)	10 (31.3%)	
Tumor number	Single	84 (77.8%)	28 (87.5%)	0.227
	Multiple	24 (22.2%)	4 (12.5%)	
Tumor differentiation	Poor	46 (42.6%)	9 (28.1%)	0.141
	Moderate-well	62 (57.4%)	23 (71.9%)	
TNM stage	0	3 (2.8%)	1 (3.1%)	0.001*
	I	7 (6.5%)	6 (18.8%)	
	II	5 (4.6%)	7 (21.9%)	
	IIIA	36 (33.3%)	9 (28.1%)	
	IIIB	37 (34.3%)	8 (25.0%)	
	IV	20 (18.5%)	1 (3.1%)	
T stage	Tis	3 (2.8%)	1 (3.1%)	<0.001*
	T1	6 (5.6%)	6 (18.8%)	
	T2	10 (9.3%)	10 (31.3%)	
	T3	82 (75.9%)	14 (43.8%)	
	T4	7 (6.5%)	1 (3.1%)	
N stage	N0	56 (51.9%)	24 (75.0%)	0.020*
	N1	43 (39.8%)	7 (21.9%)	
	N2	9 (8.3%)	1 (3.1%)	
M stage	M0	99 (91.7%)	31 (96.9%)	0.455
	M1	9 (8.3%)	1 (3.1%)	
R0 resection	No	46 (42.6%)	6 (18.8%)	0.014*
	Yes	62 (57.4%)	26 (81.3%)	
Adjuvant therapy	No	80 (74.1%)	28 (87.5%)	0.112
	Yes	28 (25.9%)	4 (12.5%)	
Jaundice	Absent	86 (79.6%)	32 (100.0%)	0.005*
	Present	22 (20.4%)	0 (0.0%)	
Gallstone	Absent	55 (50.9%)	20 (62.5%)	0.249
	Present	53 (49.1%)	12 (37.5%)	
Diabetes	Absent	84 (77.8%)	26 81.3%)	0.674
	Present	24 (22.2%)	6 (18.8%)	
Hypertension	Absent	76 (70.4%)	22 (68.8%)	0.861
	Present	32 (29.6%)	10 (31.2%)	
CA19-9 (U/mL)	≤40	39 (36.1%)	23 (71.9%)	<0.001*
	>40	69 (63.9%)	9 (28.1%)	
ALB (g/L)	≤35	20 (18.5%)	0 (0.0%)	0.009*
	>35	88 (81.5%)	32 (100.0%)	
GGT (U/L)	≤40	38 (35.2%)	32 (100.0%)	<0.001*
	>40	70 (64.8%)	0 (0.0%)	
NLR	≤1.734	25 (23.1%)	14 (43.8%)	0.022 *
	>1.734	83 (76.9%)	18 (56.2%)	
MLR	≤0.211	40 (37.0%)	23 (71.9%)	0.001*
	>0.211	68 (63.0%)	9 (28.1%)	
PLR	≤159.0	60 (55.6%)	28 (87.5%)	0.001*
	>159.0	48 (44.4%)	4 (12.5%)	
FAR	≤0.084	42 (38.9%)	24 (75.0%)	<0.001*
	>0.084	66 (61.1%)	8 (25.0%)	
Hospital stay (day)	(continuous)	16 (5-70)	11 (3-32)	<0.001*
Bleeding volume (mL)	(continuous)	200 (0-1500)	80 (10-400)	0.008*
Postsurgical complication	Absent	79 (73.1%)	30 (93.7%)	0.014*
	Present	29 (26.9%)	2 (6.3%)	

Notes: Asterisks indicate statistical significance (*P*<0.05). Abbreviations: AGR, albumin-to-γ-glutamyltransferase ratio; ALB, albumin; BMI, body mass index; CA19-9, carbohydrate antigen 19-9; FAR: fibrinogen-to-albumin ratio; GGT, γ-glutamyltransferase; MLR: monocyte-to-lymphocyte ratio; NLR: neutrophil-to-lymphocyte ratio; PLR: platelet-to-lymphocyte ratio.

**Table 2:** Univariate and multivariate analyses for OS in GBC patients.

Variable	Classification	Univariate Analysis			Multivariate Analysis		
		HR	95% CI	*P*	HR	95% CI	*P*
Sex	Male vs Female	0.870	0.576-1.313	0.507			
Age (year)	>65 vs ≤65	1.297	0.866-1.944	0.208			
BMI (kg/m^2^)	>24 vs ≤24	0.548	0.360-0.834	0.005*	0.470	0.294-0.751	0.002*
Tumor size (cm)	>3 vs ≤3	1.235	0.823-1.853	0.309			
Tumor number	Multiple vs Single	1.272	0.759-2.131	0.362			
Tumor differentiation	Poor vs moderate-well	1.663	1.105-2.502	0.015*	1.162	0.743-1.817	0.511
T stage	T3-T4 vs Tis-T2	5.798	2.990-11.242	<0.001*	3.114	1.444-6.717	0.004*
N stage	N1-N2 vs N0	2.643	1.750-3.991	<0.001*	1.119	0.656-1.911	0.680
M stage	M1 vs M0	1.319	0.608-2.865	0.483			
R0 resection	Yes vs No	0.276	0.182-0.418	<0.001*	0.448	0.265-0.758	0.003*
Adjuvant therapy	Yes vs No	1.029	0.630-1.679	0.910			
Jaundice	Present vs Absent	2.074	1.271-3.384	0.004*	0.589	0.323-1.074	0.084
Gallstone	Present vs Absent	1.042	0.698-1.557	0.840			
Diabetes	Present vs Absent	0.760	0.459-1.256	0.284			
Hypertension	Present vs Absent	0.694	0.433-1.114	0.130			
CA19-9 (U/mL)	>40 vs ≤40	3.421	2.177-5.376	<0.001*	1.704	1.005-2.892	0.048*
AGR	>2.050 vs ≤2.050	0.286	0.158-0.518	<0.001*	0.354	0.175-0.717	0.004*
NLR	>1.734 vs ≤1.734	2.988	1.778-5.022	<0.001*	1.261	0.641-2.482	0.502
MLR	>0.211 vs ≤0.211	2.387	1.566-3.638	<0.001*	0.800	0.443-1.443	0.457
PLR	>159.0 vs ≤159.0	2.324	1.547-3.489	<0.001*	1.146	0.671-1.956	0.618
FAR	>0.084 vs ≤0.084	2.720	1.776-4.164	<0.001*	1.153	0.713-1.867	0.561

Notes: Asterisks indicate statistical significance (*P*<0.05). Abbreviations: AGR, albumin-to-γ-glutamyltransferase ratio; ALB, albumin; BMI, body mass index; CA19-9, carbohydrate antigen 19-9; FAR: fibrinogen-to-albumin ratio; GGT, γ-glutamyltransferase; MLR: monocyte-to-lymphocyte ratio; NLR: neutrophil-to-lymphocyte ratio; PLR: platelet-to-lymphocyte ratio.

## Abbreviations

AGR: albumin-to-γ-glutamyltransferase ratio
AJCC: American Joint Committee on Cancer
ALB: albumin
AUC: area under ROC curve
BMI: body mass index
C index: Harrell's concordance index
CA19-9: carbohydrate antigen 19-9
CI: confidence interval
DCA: decisive curve analysis
ECOG: Eastern Cooperative Oncology Group
FAR: fibrinogen-to-albumin ratio
GBC: gallbladder cancer
GGT: γ-glutamyltransferase
HR: hazard ratio
MLR: monocyte-to-lymphocyte ratio
NLR: neutrophil-to-lymphocyte ratio
OS: overall survival
PLR: platelet-to-lymphocyte ratio
PUMCH: Peking Union Medical College Hospital
ROC: receiver operating characteristic
TNM: Tumor-Node-Metastasis
