# Supplementary material for: Prognostic Value of the Albumin-to-γ-glutamyltransferase Ratio for Gallbladder Cancer Patients and Establishing a Nomogram for Overall Survival
Source: J Cancer. 2021 May 13;12(14):4172–82. doi: 10.7150/jca.49242 (PMC8176430; doi:10.7150/jca.49242)
Supplement: Supplementary file 1 — Supplementary figures and table. [file jcav12p4172s1.pdf]

Supplementary Materials

Table S1. Baseline Characteristics

Variates	Entire cohort (n=140)	Training cohort (n=80)	Validation cohort (n=60)	*P*
**Sex**				0.276
Female	82 (58.6%)	50 (62.5%)	32 (53.3%)	
Male	58 (41.4%)	30 (37.5%)	28 (46.7%)	
**Age**				0.200
≤65	80 (57.1%)	42 (52.5%)	38 (63.3%)	
>65	60 (42.9%)	38 (47.5%)	22 (36.7%)	
**BMI (kg/m^2)**				0.590
≤24	76 (54.3%)	45 (56.2%)	31 (51.7%)	
>24	64 (45.7%)	35 (43.8%)	29 (48.3%)	
**Tumor size (cm)**				0.155
≤3	81 (57.9%)	59 (54.6%)	22 (68.8%)	
>3	59 (42.1%)	49 (45.4%)	10 (31.3%)	
**Tumor number**				0.393
Single	112 (80.0%)	62 (77.5%)	50 (83.3%)	
Multiple	28 (20.0%)	18 (22.5%)	10 (16.7%)	
**Tumor differentiation**				0.231
Poor	55 (39.3%)	28 (35.0%)	27 (45.0%)	
Moderate-well	85 (60.7%)	52 (65.0%)	33 (55.0%)	
**TNM stage**				0.529
0	4 (2.9%)	2 (2.5%)	2 (3.3%)	
I	13 (9.3%)	10 (12.5%)	3 (5.0%)	
II	12 (8.6%)	6 (7.5%)	6 (10.0%)	
IIIA	45 (32.1%)	26 (32.5%)	19 (31.7%)	
IIIB	45 (32.1%)	24 (30.0%)	21 (35.0%)	
IV	21 (15.0%)	12 (15.0%)	9 (15.0%)	
**T stage**				0.615
Tis	4 (2.9%)	2 (2.5%)	2 (3.3%)	
T1	12 (8.6%)	10 (12.5%)	2 (3.3%)	
T2	20 (14.3%)	9 (11.3%)	11 (18.3%)	
T3	96 (68.6%)	55 (68.8%)	41 (68.3%)	
T4	8 (5.7%)	4 (5.0%)	4 (6.7%)	
**N stage**				0.336
N0	80 (57.1%)	48 (60.0%)	32 (53.3%)	
N1	50 (35.7%)	28 (35.0%)	22 (36.7%)	
N2	10 (7.1%)	4 (5.0%)	6 (10.0%)	
**M stage**				0.745
M0	130 (92.9%)	75 (93.8%)	55 (91.7%)	
M1	10 (7.1%)	5 (6.3%)	5 (8.3%)	
**R0 resection**				0.130
No	52 (37.1%)	34 (42.5%)	18 (30.0%)	
Yes	88 (62.9%)	46 (57.5%)	42 (70.0%)	
**Adjuvant therapy**				0.486
No	108 (77.1%)	60 (75.0%)	48 (80.0%)	
Yes	32 (22.9%)	20 (25.0%)	12 (20.0%)	
**Jaundice**				0.841
No	118 (84.29%)	67 (83.8%)	51 (85.0%)	
Yes	22 (15.71%)	13 (16.2%)	9 (15.0%)	
**Gallstone**				0.007*
No	75 (53.57%)	35 (43.8%)	40 (66.7%)	
Yes	65 (46.43%)	45 (56.2%)	20 (33.3%)	
**Diabetes**				0.634

No	110 (78.57%)	64 (80.0%)	46 (76.7%)	
Yes	30 (21.43%)	16 (20.0%)	14 (23.3%)	
**Hypertension**				0.001*
No	98 (70.0%)	47 (58.8%)	51 (85.0%)	
Yes	42 (30.0%)	33 (41.3%)	9 (15.0%)	
**CA19-9 (U/mL)**				0.883
≤40	62 (44.3%)	35 (43.8%)	27 (45.0%)	
>40	78 (55.7%)	45 (56.2%)	33 (55.0%)	
**ALB (g/L)**				0.486
≤35	20 (14.3%)	10 (12.5%)	10 (16.7%)	
>35	120 (85.7%)	70 (87.5%)	50 (83.3%)	
**GGT (U/L)**				0.495
≤40	70 (50.00%)	42 (52.5%)	28 (46.7%)	
>40	70 (50.00%)	38 (47.5%)	32 (53.3%)	
**AGR**				0.771
≤2.050	108 (77.1%)	61 (76.3%)	47 (78.3%)	
>2.050	32 (22.9%)	19 (23.8%)	13 (21.7%)	
**NLR**				0.514
≤1.734	39 (27.9%)	24 (30.0%)	15 (25.0%)	
>1.734	101 (72.1%)	56 (70.0%)	45 (75.0%)	
**MLR**				0.170
≤0.211	63 (45.0%)	40 (50.0%)	23 (38.3%)	
>0.211	77 (55.0%)	40 (50.0%)	37 (61.7%)	
**PLR**				0.801
≤159.0	88 (62.9%)	51 (63.7%)	37 (61.7%)	
>159.0	52 (37.1%)	29 (36.3%)	23 (38.3%)	
**FAR**				0.261
≤0.084	66 (47.1%)	41 (51.2%)	25 (41.7%)	
>0.084	74 (52.9%)	39 (48.8%)	35 (58.3%)	
**Hospital stay (day)**	15 (3–70)	15 (3–68)	15 (5–70)	0.239
**Bleeding volume (mL)**	200 (0–1500)	200 (0–800)	200 (0–1500)	0.485
**Postsurgical complication**				0.481
No	109 (77.9%)	64 (80.0%)	45 (75.0%)	
Yes	31 (22.1%)	16 (20.0%)	15 (25.0%)	

Notes: Asterisks indicate statistically significant differences between the training cohort and the validation cohort (P<0.05).

Abbreviations: AGR, albumin-to-γ-glutamyltransferase ratio; ALB, albumin; BMI, body mass index; CA19-9, carbohydrate antigen 19-9; FAR: fibrinogen-to-albumin ratio; GGT, γ-glutamyltransferase; MLR: monocyte-to-lymphocyte ratio; NLR: neutrophil-to-lymphocyte ratio; PLR: platelet-to-lymphocyte ratio.

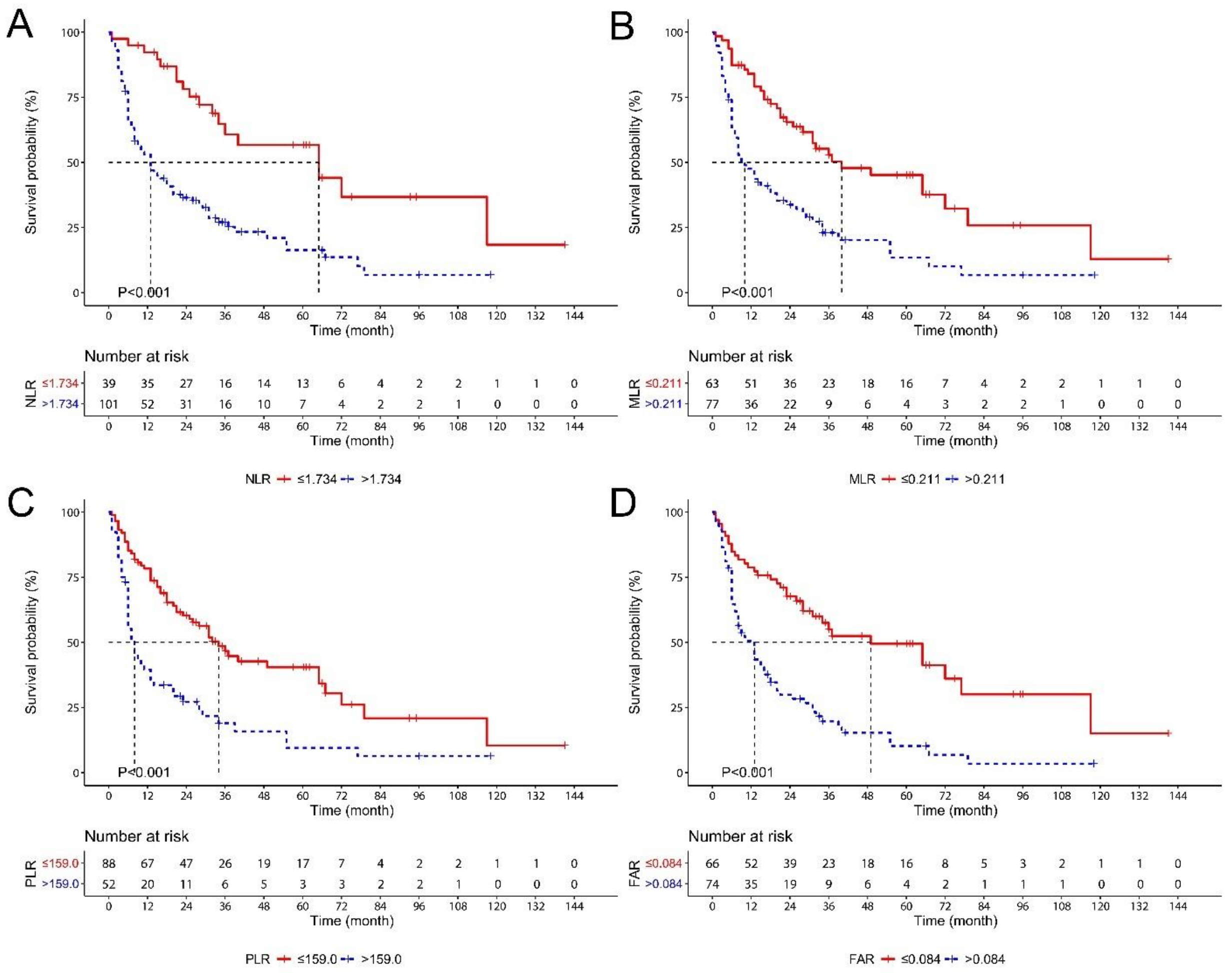

Figure S1. Kaplan-Meier survival curves for overall survival in gallbladder cancer patients stratified according to NLR (A), MLR (B), PLR (C) and FAR (D).

Abbreviations: FAR, fibrinogen-to-albumin ratio; MLR, monocyte-to-lymphocyte ratio; NLR, neutrophil-to-lymphocyte ratio; PLR, platelet-to-lymphocyte ratio.

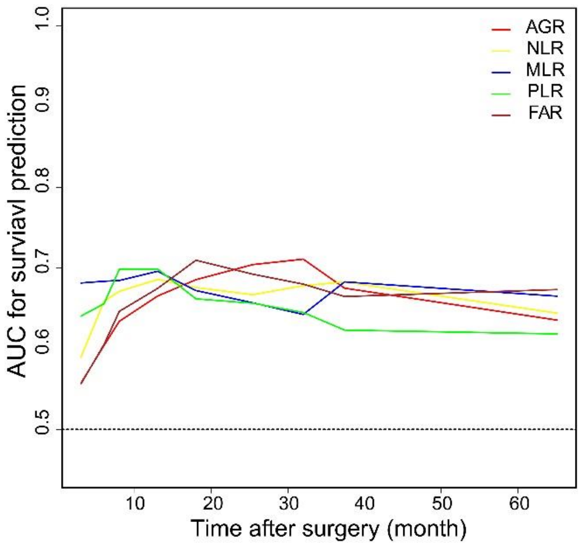

Figure S2. Time-dependent area under ROC curves of AGR and other inflammation-related indies for overall survival prediction**.**

Abbreviations: AGR, albumin-to-γ-glutamyltransferase ratio; FAR, fibrinogen-to-albumin ratio; MLR, monocyte-to-lymphocyte ratio; NLR, neutrophil-to-lymphocyte ratio; PLR, platelet-to-lymphocyte ratio.

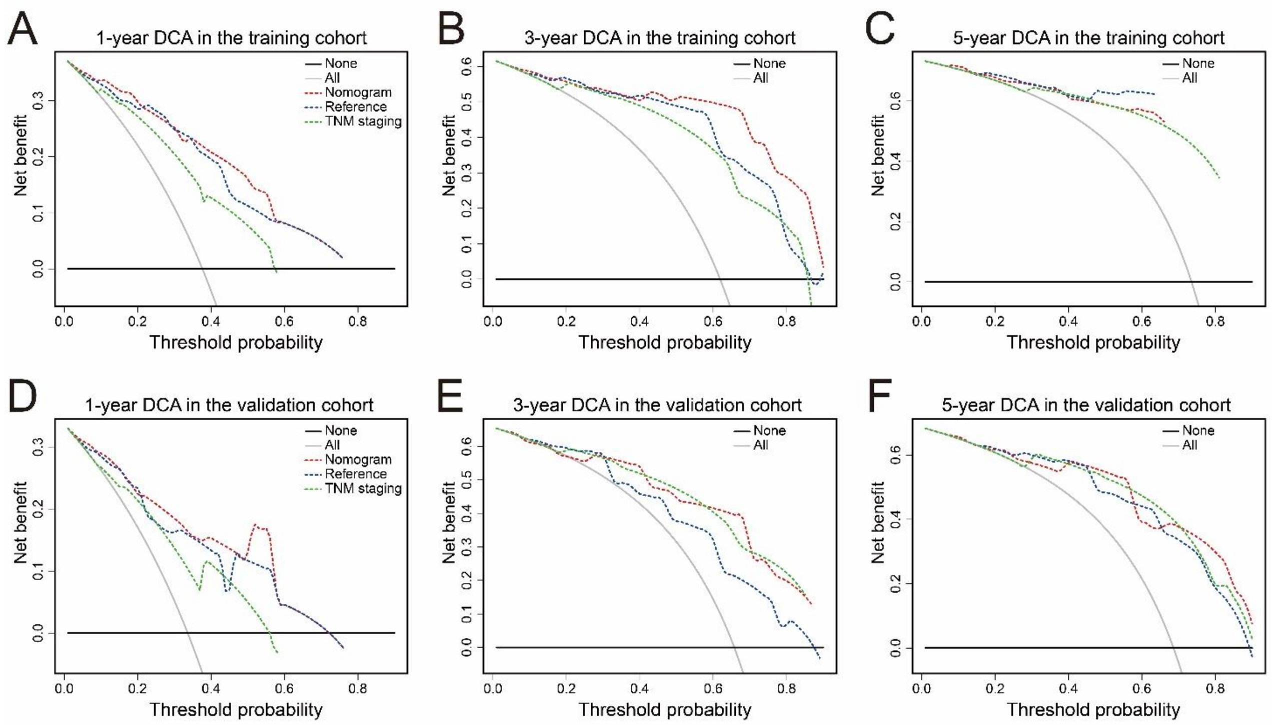

Figure S3. Decisive curve analyses of the nomogram (red), the reference model (blue) and the TNM staging system (green) for 1-, 3- and 5-year overall survival in the training cohort (A-C) and the validation cohort (D-F). The horizontal black line represents all patients are negative and the net benefit is 0; the grey line represents all patients are positive. The time was marked on the top of each panel.

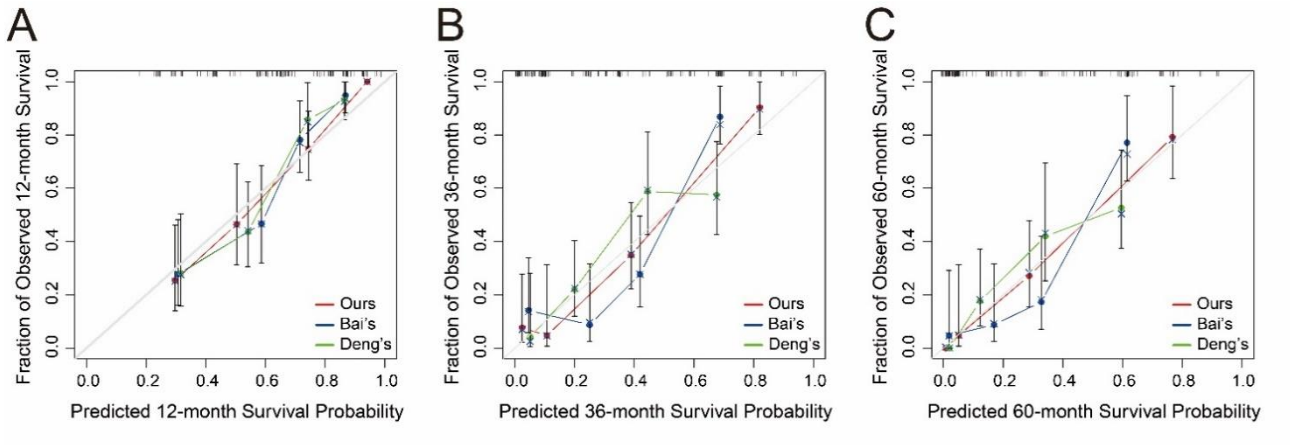

Figure S4. Calibration curves of our (red), Bai's (blue) and Deng's (green) nomograms for 1-, 3- and 5-year overall survival. The x-axis represents nomogram predicted probability of survival, and the y-axis is the actually observed survival probability.

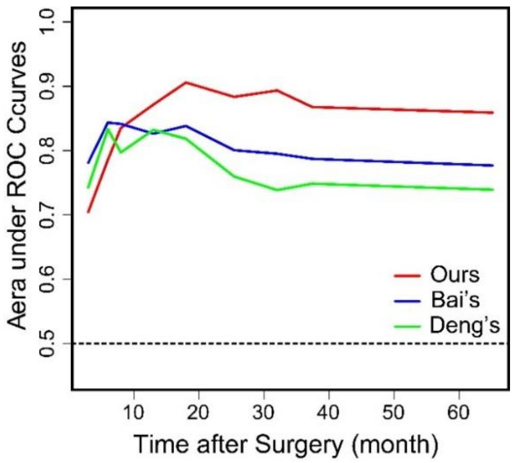

Figure S5. Time-dependent area under ROC curves of our (red), Bai's (blue) and Deng's (green) nomograms.

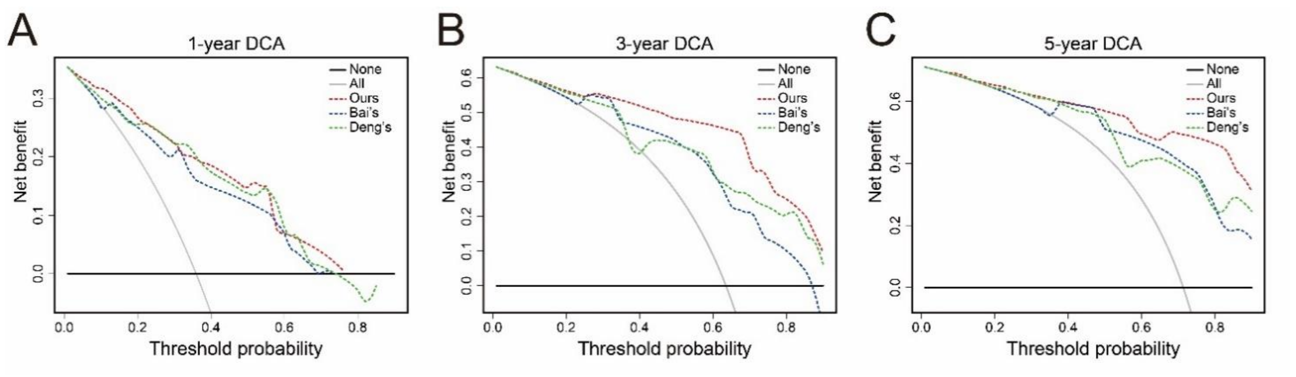

Figure S6. Decisive curve analysis plots of our (red), Bai's (blue) and Deng's (green) nomograms for 1-, 3- and 5-year overall survival. The horizontal black line represents all patients are negative and the net benefit is 0; the grey line represents all patients are positive. The time was marked on the top of each panel.

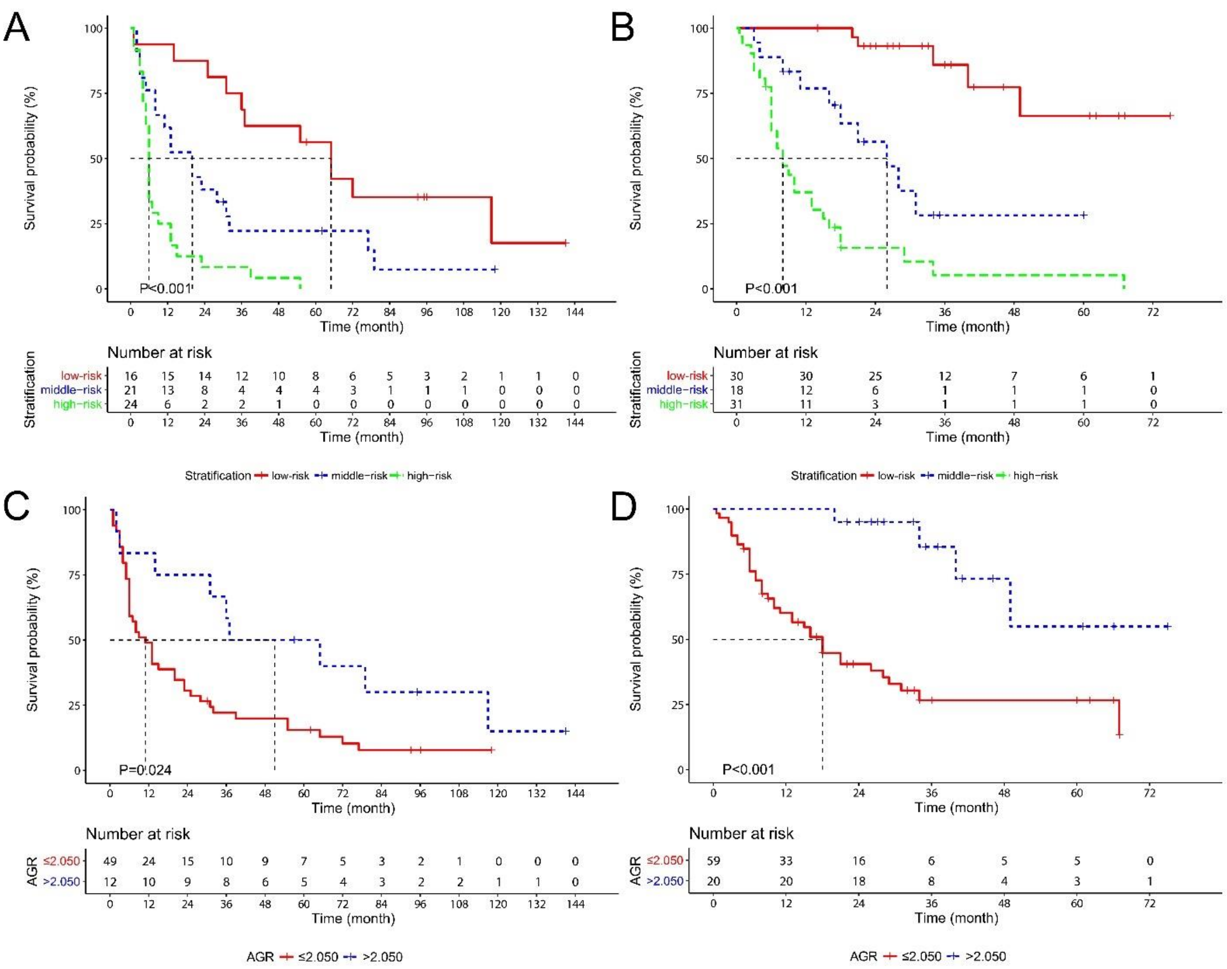

Figure S7. Kaplan-Meier survival curves for overall survival according to the risk stratification model (A-B) and AGR (C-D) in the 2003-2012 and 2013-2017 subgroups. A: survival curves of the risk stratification model in the 2003-2012 subgroup; B: survival curves of the risk stratification model in the 2013-2017 subgroup; C: survival curves of different levels of AGR in the 2003-2012 subgroup; D: survival curves of different levels of AGR in the 2013-2017 subgroup.

Abbreviations: AGR, albumin-to-γ-glutamyltransferase ratio.

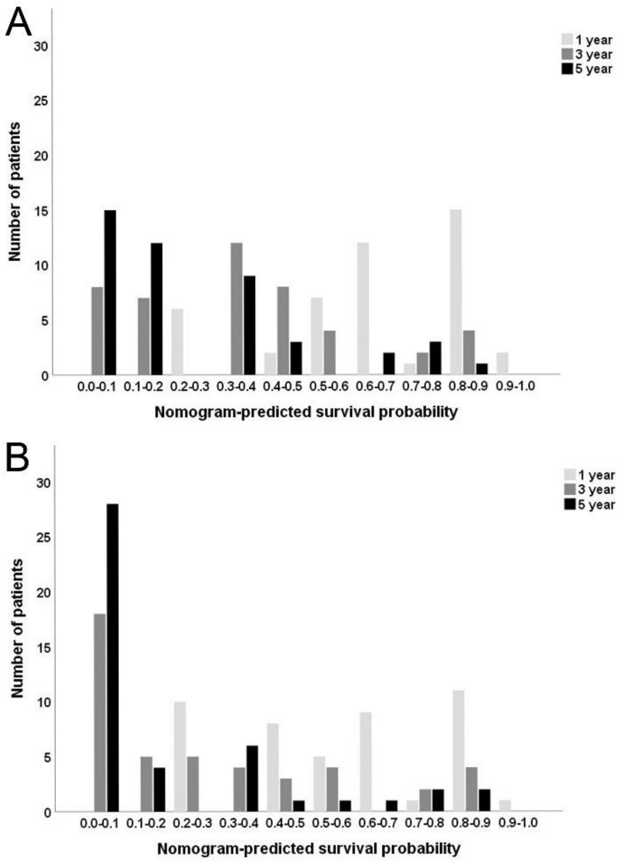

Figure S8. Survival probability predicted by nomogram in patients with TNM stage IIIA (A) & IIIB (B). The x-axis represents the nomogram-predicted survival probability at 1, 3 and 5 years after surgery, and the y-axis is the number of patients within the corresponding interval of nomogram-predicted survival probability.
